# Exclusive and dual influenza and COVID-19 vaccination among U.S. adults and adolescents in 2021

**DOI:** 10.1080/07853890.2023.2196436

**Published:** 2023-04-13

**Authors:** Kimberly H. Nguyen, Ruitong Zhao, Siyu Chen, Robert A. Bednarczyk

**Affiliations:** a Department of Public Health & Community Medicine, Tufts University School of Medicine, Boston, MA, USA; b Hubert Department of Global Health, Emory University Rollins School of Public Health, Atlanta, GA, USA; c Department of Epidemiology, Emory University Rollins School of Public Health, Atlanta, GA, USA; d Emory Vaccine Center, Emory University, Atlanta, GA, USA

**Keywords:** Vaccine hesitancy, influenza, COVID-19, adults, adolescents, access, barriers, exclusive, dual vaccination, pandemic, United States

## Abstract

**Introduction:**

Despite recommendations for influenza and COVID-19 vaccines, studies have documented gaps and disparities in vaccination coverage for adults and adolescents. Understanding the proportion and demographics of those unvaccinated against influenza and/or COVID-19 is important for tailoring appropriate messaging and strategies to increase confidence and uptake.

**Methods:**

Using the 2021 National Health Interview Survey (NHIS), we assessed the prevalence of four vaccination patterns (exclusive influenza vaccination, exclusive COVID-19 vaccination, dual influenza and COVID-19 vaccination, and neither vaccination) by sociodemographic and other characteristics among adults and adolescents 12–17 years. Adjusted multivariable regression analyses were conducted to examine factors associated with each of the four vaccination categories among adults and adolescents.

**Results:**

In 2021, 42.5% of adults and 28.3% of adolescents received both influenza and COVID-19 vaccines, while approximately a quarter (22.4%) of adults and a third (34.0%) of adolescents did not receive either vaccine. Among adults and adolescents, 6.0% and 11.4% were exclusively vaccinated against influenza and 29.1% and 26.4% were exclusively vaccinated against COVID-19, respectively. Among adults, exclusive COVID-19 or dual vaccination was more likely to be associated with older age, non-Hispanic multi/other race, and having a college degree compared to their respective counterparts. Exclusive influenza or neither vaccination was more likely to be associated with younger age, having a high school diploma or less, living below the poverty level, and having a previous COVID-19 diagnosis.

**Conclusion:**

During the COVID-19 pandemic, approximately two-thirds of adolescents and three-fourths of adults received exclusive influenza or COVID-19 vaccines or both vaccines in 2021. Vaccination patterns differed by sociodemographic and other characteristics. Promoting confidence in vaccines and reducing barriers to access is needed to protect individuals and families from severe health consequences of vaccine-preventable diseases. Being up-to-date with all recommended vaccinations can prevent a future resurgence of hospitalizations and cases.

**Key messages**
- 42.5% of adults and 28.3% of adolescents received both influenza and COVID-19 vaccines in 2021, while approximately a quarter (22.4%) of adults and a third (34.0%) of adolescents did not receive either vaccine; 6.0% of adults and 11.4% of adolescents were exclusively vaccinated against influenza and 29.1% of adults and 26.4% of adolescents were exclusively vaccinated against COVID-19.
- Among adults, exclusive COVID-19 vaccination or dual vaccination was more likely to be associated with older age, non-Hispanic multi/other race, and having a college degree or higher compared to their respective counterparts; exclusive influenza vaccination or neither vaccination was more likely to be associated with younger age, having a high school diploma or less, living below poverty level, and having a previous COVID-19 diagnosis compared to their respective counterparts.
- Promoting confidence in vaccines and reducing barriers to access is needed to protect individuals and families from severe health consequences of vaccine-preventable diseases. Being up-to-date with all recommended vaccinations can prevent a future resurgence of hospitalizations and cases, especially as new variants emerge.

## Introduction

The Advisory Council for Immunization Practices (ACIP) recommends influenza vaccinations for everyone ages 6 months and older, and in December 2020, recommended COVID-19 vaccinations for adults 18 years and older in the U.S. (and some adolescents ages 16 and older depending on the vaccine) [1,2]. COVID-19 vaccinations were recommended for all adolescents 12–17 years in May 2021. Being vaccinated against both influenza and COVID-19 is needed to protect people against vaccine-preventable diseases and prevent severe outcomes such as hospitalizations and deaths. In addition, it can prevent a future “twindemic” or “tripledemic” caused by high cases of COVID-19, influenza, or other respiratory viruses, that could overwhelm an already strained healthcare system [3].

Despite these recommendations, studies have documented gaps and disparities in influenza vaccination coverage [4,5]. In 2018 and 2019, influenza coverage was 46.1% among adults and 52.2% among adolescents 13–17 years [6,7]. Studies noted that the main reasons for not being vaccinated against influenza are the belief that one is unlikely to get very sick from influenza or will never get influenza [8]. Other barriers such as access and cost may also contribute to the gaps in vaccination coverage [9]. While trends for influenza vaccination coverage increased slightly for adults from 38.3% in the 2010–2011 season to 43.4% in the 2015–2016 season, it did not significantly increase for adolescents from 2012–2018 [7,10].

With the onset of the COVID-19 pandemic, similar disparities were present for adults and adolescents [11–13]. In 2021, COVID-19 vaccines were offered free of charge at mass vaccination sites and healthcare providers’ offices [14]. Yet, numerous studies have documented hesitancy toward the COVID-19 vaccine for adults and adolescents. While COVID-19 vaccination coverage (≥1 dose) was 73.6% for adults and 64.2% for adolescents 12–17 years by 31 December 2021, those who were unvaccinated reported concerns about potential side effects from the vaccine, lack of trust in the vaccines, or a desire to wait and get it later for themselves or for their adolescent children [15–18]. Similar to the influenza vaccine, barriers to access may include logistical or transportation issues, which could have prevented many people from getting access to COVID-19 primary series or boosters [19,20].

Because there are gaps in coverage for both influenza and COVID-19 vaccines, understanding the distribution and sociodemographic characteristics of those who are unvaccinated against influenza and/or COVID-19 is important for tailoring appropriate messaging and strategies to increase confidence and uptake of these recommended vaccines [21–23]. It is unclear whether those who are unvaccinated against COVID-19 are also unvaccinated against influenza, and the factors that are associated with each, both, or neither vaccinations. This study examined the distribution and sociodemographic characteristics of adults and adolescents who received exclusive influenza or COVID-19 vaccinations, dual vaccinations, or neither influenza nor COVID-19 vaccinations in 2021 using a large, nationally representative sample of households in the U.S.

## Methods

### Study sample

The National Health Interview Survey (NHIS) is a cross-sectional nationally representative household survey of U.S. adults that is conducted throughout the year by the U.S. Census Bureau for Center for Disease Control and Prevention (CDC) National Center for Health Statistics. Using a complex sampling design, the NHIS sample was selected using stratification, clustering, and multistage sampling, and estimates were weighted to the adult civilian noninstitutionalized population of the U.S. population to minimize non-response bias. In-person interviews are conducted in a probability sample of households. For the adult questionnaire, one adult aged ≥18 years is randomly selected to complete the survey within each family in the household. For the child questionnaire, one child is randomly selected from each family. A knowledgeable adult, most often the parent or guardian of the child, responds on behalf of the child. In 2021, COVID-19 vaccination questions were added to the NHIS. The entire questionnaire is conducted face-to-face. This study is a secondary analysis of data collected for adults and adolescents ages 12–17 years. COVID-19 vaccination was not recommended for children aged 5–11 years until November 2021, so this age group was not included in the study [2]. The adult sample size and the response rate was 29,482 and 50.9%, respectively, and the adolescent sample size and the response rate were 3,207 and 49.9%, respectively [24]. This study was reviewed by the Tufts University Health Sciences Institutional Review Board and determined not to be human subjects research (Study ID: 00003092).

### Measures

Influenza vaccination status was assessed by asking respondents whether they (or the adolescent) had received the influenza vaccine during the past 12 months. COVID-19 vaccination status was assessed by asking respondents whether they (or the adolescent) had received the COVID-19 vaccine. Vaccination measures included those who were vaccinated against influenza but not against COVID-19 (exclusive influenza vaccination), those who were vaccinated against COVID-19 but not against influenza (exclusive COVID-19 vaccination), those who were vaccinated against both influenza and COVID-19 (dual vaccination), and those who were vaccinated against neither influenza nor COVID-19 (neither vaccination).

People with high-risk conditions are at increased risk for severe complications from influenza and COVID-19 [25]. Adults who are at high-risk were defined as those who reported one or more of the following: ever being told by a physician they had diabetes, emphysema, chronic obstructive pulmonary disease, coronary heart disease, angina, heart attack, or other heart condition; being diagnosed with cancer in the past 12 months (excluding nonmelanoma skin cancer) or ever being told by a physician they had lymphoma, leukemia, or blood cancer; during the past 12 months, being told by a physician they had chronic bronchitis or weak or failing kidneys; or reporting an asthma episode or attack in the past 12 months [25]. Adolescent high-risk status was based on parental report of 1 or more of the following: diabetes or reporting an asthma episode or attack in the past 12 months (i.e. current asthma). While this list may represent the majority of people with high-risk medical conditions based on ACIP recommendations, there may be a few other high-risk conditions such as cystic fibrosis, sickle cell anemia, congenital heart disease, cerebral palsy, and other conditions that are not listed here due to the limitations of the survey.

Sociodemographic variables assessed for adults were age, sex, race/ethnicity, educational status, marital status, poverty level, and region. Sociodemographic variables assessed for adolescents were age, sex, race/ethnicity, poverty level, and region. COVID-19 diagnosis was defined as being told by a doctor or health professional that you (or the adolescent) had or likely had coronavirus or COVID-19. Healthcare personnel (HCP) were defined as adults aged ≥18 years who reported they currently volunteered or worked in a hospital, medical clinic, doctor’s office, dentist’s office, nursing home, or some other healthcare facility, including part-time and unpaid work in a healthcare facility as well as professional nursing care provided in the home. Other variables assessed included health insurance, having a usual place for healthcare and the number of hospitalizations within the past year.

### Statistical analysis

Weighted point estimates and 95% confidence intervals (CIs) were calculated using SAS version 9.4 to account for complex sample survey design and to produce nationally representative estimates. Sociodemographic characteristics were assessed for adults and adolescents aged 12–17 years. In bivariate analyses, the prevalence of the four vaccination categories was assessed by sociodemographic and other characteristics for adults and adolescents. Adjusted multivariable regression analyses were conducted to examine factors associated with each of the four vaccination categories. All tests were two-tailed with the significance level set at *α* < 0.05. All results presented in the text of this manuscript are statistically significant unless otherwise noted.

## Results

The proportion of adults within each age group and sex was almost evenly distributed, with slightly higher percentages of adults who were 50–64 years (24.6%) and ≥65 years (22.1%) (Table 1). The majority of adults were Non-Hispanic (NH) White (62.8%), had a high school diploma or less (37.8%), lived at or above the poverty level (90.1%), married or lived with a partner (61.1%), and lived in the South (37.9%). Approximately 90% had health insurance and a usual place for healthcare, and 8% were hospitalized in the past year. Over a quarter of adults (26.0%) had a high-risk condition, and 13.5% had a previous COVID-19 diagnosis.

**Table 1.** Distribution of demographic and other characteristics among adults, National Health Interview Survey, United States, 2021.

	Weighted percent (95% CI)
Overall (n)	29,482
Age group (years)	
18–29	20.2 (19.5, 21.0)
30–39	17.1 (16.5, 17.6)
40–49	16.0 (15.5, 16.5)
50–64	24.6 (24.0, 25.2)
≥65	22.1 (21.5, 22.8)
Sex	
Male	48.3 (47.6, 49.0)
Female	51.7 (51.0, 52.4)
Race/ethnicity	
NH White	62.8 (61.2, 64.3)
NH Black	11.7 (10.8, 12.6)
NH multi/other	8.6 (7.9, 9.4)
Hispanic or Latino	16.9 (15.6, 18.2)
Educational status	
High school or less	37.8 (36.8, 38.8)
Some college and Associate degree	26.6 (25.9, 27.3)
College graduate (Bachelor’s degree)	22.5 (21.8, 23.2)
Above college graduate	13.1 (12.5, 13.7)
Poverty level	
At or above	90.1 (89.5, 90.7)
Below	9.9 (9.3, 10.5)
Marital status	
Married or living with a partner	61.1 (60.3,61.8)
Widowed/divorced/separated	14.8 (14.3,15.2)
Never married	24.1 (23.4, 24.9)
Region	
Northeast	17.5 (16.3, 18.6)
Midwest	20.8 (19.6, 22.0)
South	37.9 (36.4, 39.5)
West	23.8 (22.3, 25.3)
Health Insurance	
Yes	90.0 (89.5, 90.6)
No	10.0 (9.4, 10.5)
The usual place for healthcare	
Yes	89.6 (89.1, 90.1)
No	10.4 (9.9, 10.9)
Hospitalizations within the past year	
yes	8.2 (7.9, 8.6)
No	91.8 (91.4, 92.1)
High-risk condition^a^	
Yes	26.0 (25.4, 26.6)
No	74.0 (73.4, 74.6)
Previous COVID-19 diagnosis	
Yes	13.5 (13.0, 14.1)
No	86.5 (85.9, 87.0)
Healthcare provider^b^	
Yes	3.3 (3.0, 3.6)
No	96.7 (96.4, 97.0)

NH: Non-Hispanic; CI: confidence interval.

^a^Adults categorized as being at high risk for influenza-related complications reported one or more of the following: (1) ever being told by a physician that they had diabetes, emphysema, chronic obstructive pulmonary disease, coronary heart disease, angina, heart attack, or another heart condition; (2) receiving a diagnosis of cancer during the preceding 12 months (excluding nonmelanoma skin cancer) or ever being told by a physician that they had lymphoma, leukemia, or blood cancer; (3) being told by a physician that they had chronic bronchitis or weak or failing kidneys during the preceding 12 months; or (4) reporting an asthma episode or attack during the preceding 12 months.

^b^Adults were classified as healthcare personnel if they reported that they currently volunteer or work in a hospital, medical clinic, doctor’s office, dentist’s office, nursing home, or some other healthcare facility, including part-time and unpaid work in a healthcare facility as well as professional nursing care provided in the home.

**Figure 1.**
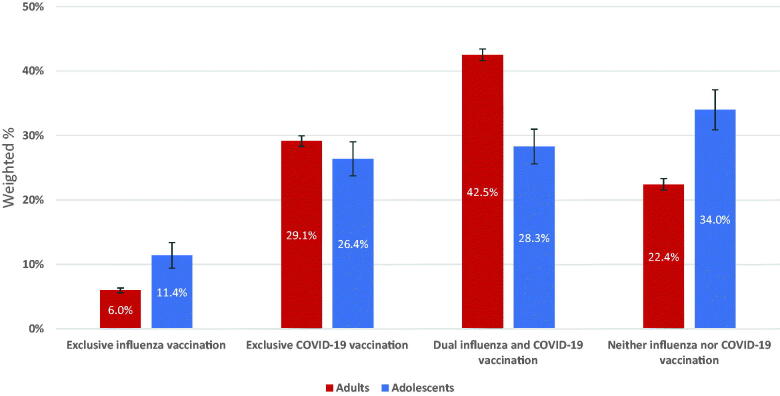
Distribution of Influenza and/or COVID-19 vaccination coverage among adults and adolescents 12–17 years, National Health Interview Survey, United States, 2021.

Among adults, 6.0% were exclusively vaccinated against influenza, 29.1% were exclusively vaccinated against COVID-19, 42.5% were vaccinated against both, and 22.4% were vaccinated against neither (Table 2, Figure 1). Exclusive influenza vaccination was highest among young adults (18–29 years: adjusted prevalence ratio [aPR] = 2.13), females (aPR = 1.27), those with a high school diploma or less (aPR = 3.23), and those living below the poverty level (aPR = 1.39). Those who were hospitalized within the past year (aPR = 1.73) and had a previous COVID-19 diagnosis (aPR = 1.39) were more likely to be exclusively vaccinated against influenza than those who were not hospitalized in the past year and those who did not have a previous COVID-19 diagnosis, respectively. Adults who were Hispanic (aPR = 0.76) and NH other/multiple races (aPR = 0.69) compared to NH White adults, or did not have a usual place for healthcare (aPR = 0.72) compared to those who did have a usual place for healthcare, were less likely to be exclusively vaccinated against influenza (Table 2).

**Table 2.** Bivariate and multivariable analyses of adults’ receipt of influenza and/or COVID-19 vaccination, National Health Interview Survey, United States, 2021.

	Bivariate analysis				Multivariable analysis			
	Exclusive influenza vaccination	Exclusive COVID-19 vaccination	Dual influenza and COVID-19 vaccination	Neither influenza nor COVID-19 vaccination	Exclusive influenza vaccination	Exclusive COVID-19 vaccination	Dual influenza and COVID-19 vaccination	Neither influenza nor COVID-19 vaccination
	% (95%CI)	% (95%CI)	% (95%CI)	% (95%CI)	aPR (95% CI)*	aPR (95% CI)*	aPR (95% CI)*	aPR (95% CI)*
Overall	6.0 (5.6, 6.4)	29.1 (28.3, 29.9)	42.5 (41.6, 43.5)	22.4 (21.5, 23.3)				
Age group (years)								
18–29	7.3 (6.2, 8.4)	31.1 (29.0, 33.2)	26.4 (24.4, 28.4)	35.2 (32.9, 37.5)	2.13 (1.59, 2.85)	1.41 (1.28, 1.56)	Ref	3.84 (3.36, 4.39)
30–39	7.2 (6.1, 8.3)	32.2 (30.4, 34.0)	30.1 (28.2, 32.0)	30.5 (28.6, 32.4)	2.13 (1.64, 2.77)	1.54 (1.40, 1.69)	1.03 (0.92, 1.15)	3.66 (3.19, 4.20)
40–49	5.6 (4.6, 6.6)	32.8 (30.9, 34.8)	34.9 (32.8, 36.9)	26.7 (24.8, 28.5)	1.62 (1.30, 2.02)	1.52 (1.41, 1.64)	1.22 (1.11, 1.34)	3.32 (2.92, 3.77)
50–64	5.6 (4.8, 6.3)	31.6 (30.2, 33.1)	46.4 (44.8, 48.0)	16.4 (15.3, 17.6)	1.53 (1.24, 1.89)	1.56 (1.45, 1.68)	1.60 (1.47, 1.74)	1.96 (1.72, 2.23)
≥65	4.5 (3.9, 5.1)	19.7 (18.5, 20.8)	67.6 (66.2, 69.0)	8.2 (7.4, 9.0)	Ref	Ref	2.24 (2.06, 2.44)	Ref
Sex								
Male	5.0 (4.5, 5.6)	31.5 (30.3, 32.6)	39.2 (38.0, 40.5)	24.3 (23.1, 25.6)	Ref	Ref	Ref	Ref
Female	6.9 (6.3, 7.4)	27.0 (26.0, 27.9)	45.6 (44.3, 46.8)	20.6 (19.6, 21.6)	1.27 (1.10, 1.46)	0.90 (0.86, 0.93)	1.09 (1.06, 1.13)	0.96 (0.91, 1.02)
Race/ethnicity								
NH White	6.0 (5.5, 6.5)	26.3 (25.4, 27.1)	46.6 (45.6, 47.7)	21.1 (20.1, 22.0)	Ref	Ref	Ref	Ref
NH Black	6.7 (5.3, 8.1)	33.2 (30.8, 35.7)	32.0 (29.5, 34.5)	28.0 (25.6, 30.4)	0.82 (0.66, 1.01)	1.24 (1.14, 1.36)	0.85 (0.79, 0.92)	0.99 (0.89, 1.09)
NH multi/other	4.0 (3.1, 5.0)	33.3 (30.8, 35.8)	48.2 (45.4, 51.0)	14.5 (12.3, 16.7)	0.69 (0.53, 0.89)	1.22 (1.13, 1.31)	1.11 (1.05, 1.18)	0.67 (0.59, 0.76)
Hispanic or Latino	6.1 (5.2, 7.1)	34.9 (32.6, 37.2)	31.4 (29.5, 33.4)	27.5 (25.3, 29.7)	0.76 (0.64, 0.90)	1.28 (1.18, 1.39)	1.00 (0.93, 1.08)	0.78 (0.70, 0.86)
Educational Status								
High school or less	8.0 (7.2, 8.7)	27.4 (26.0, 28.7)	32.8 (31.5, 34.1)	31.9 (30.4, 33.4)	3.23 (2.35, 4.44)	0.92 (0.85, 0.99)	Ref	3.65 (3.17, 4.20)
Some college and Associate degree	6.7 (5.9, 7.5)	30.0 (28.5, 31.4)	38.9 (37.3, 40.4)	24.5 (23.1, 25.9)	2.54 (1.83, 3.53)	1.06 (0.99, 1.14)	1.09 (1.03, 1.15)	2.93 (2.52, 3.40)
College graduate (Bachelor degree)	3.9 (3.3, 4.6)	32.3 (30.7, 33.9)	50.8 (49.1, 52.6)	13.0 (11.9, 14.1)	1.60 (1.16, 2.21)	1.13 (1.05, 1.20)	1.43 (1.35, 1.51)	1.59 (1.35, 1.88)
Above college graduate	2.5 (1.9, 3.2)	27.1 (25.4, 28.9)	63.3 (61.3, 65.3)	7.1 (6.0, 8.1)	Ref	Ref	1.61 (1.53, 1.70)	Ref
Poverty level								
At or above (ref)	5.5 (5.1, 5.9)	29.3 (28.4, 30.2)	44.2 (43.2, 45.2)	21.0 (20.1, 21.8)	Ref	Ref	1.29 (1.17, 1.41)	Ref
Below	10.0 (8.4, 11.6)	27.5 (25.2, 29.9)	26.8 (24.4, 29.2)	35.7 (33.0, 38.4)	1.39 (1.13, 1.69)	0.94 (0.85, 1.03)	Ref	1.23 (1.13, 1.34)
Marital status								
Married	5.6 (5.1, 6.1)	28.2 (27.2, 29.2)	46.0 (44.8, 47.1)	20.2 (19.2, 21.2)	Ref	Ref	Ref	Ref
Widowed/divorced/separated	6.5 (5.6, 7.4)	26.7 (25.3, 28.2)	48.1 (46.3, 49.8)	18.7 (17.3, 20.1)	1.18 (0.96, 1.44)	1.09 (1.01, 1.17)	0.90 (0.86, 0.94)	1.20 (1.09, 1.32)
Never married	6.3 (5.4, 7.3)	33.1 (31.3, 35.0)	30.3 (28.5, 32.1)	30.2 (28.3, 32.2)	0.91 (0.71, 1.15)	1.08 (1.01, 1.15)	1.01 (0.96, 1.07)	0.91 (0.85, 0.98)
Region								
Northeast	5.1 (4.2, 6.1)	30.3 (28.2, 32.4)	48.3 (46.0, 50.6)	16.2 (14.4, 18.1)	Ref	Ref	Ref	Ref
Midwest	6.3 (5.4, 7.2)	26.8 (25.4, 28.2)	44.2 (42.4, 46.1)	22.7 (21.1, 24.2)	1.05 (0.85, 1.31)	0.92 (0.83, 1.02)	0.97 (0.91, 1.04)	1.21 (1.06, 1.39)
South	6.7 (6.0, 7.4)	28.3 (26.9, 29.8)	38.4 (36.7, 40.0)	26.6 (25.0, 28.3)	1.13 (0.94, 1.35)	0.90 (0.82, 0.99)	0.89 (0.83, 0.94)	1.41 (1.24, 1.60)
West	5.1 (4.4, 5.9)	31.5 (30.0, 33.1)	43.3 (41.5, 45.2)	20.0 (18.3, 21.7)	1.01 (0.80, 1.27)	0.97 (0.88, 1.08)	0.97 (0.92, 1.02)	1.13 (0.98, 1.30)
Health insurance								
Yes	5.9 (5.4, 6.3)	28.5 (27.7, 29.3)	45.9 (44.9, 46.8)	19.8 (19.0, 20.7)	Ref	Ref	Ref	Ref
No	6.8 (5.4, 8.3)	35.4 (32.5, 38.4)	12.3 (10.6, 14.1)	45.4 (42.4, 48.5)	0.97 (0.77, 1.21)	1.05 (0.93, 1.17)	0.43 (0.37, 0.51)	1.29 (1.17, 1.43)
Usual place for healthcare								
Yes	6.1 (5.7, 6.5)	28.3 (27.5, 29.1)	45.5 (44.5, 46.5)	20.1 (19.3, 21.0)	Ref	Ref	Ref	Ref
No	4.7 (3.4, 5.9)	36.6 (33.8, 39.5)	16.2 (14.2, 18.2)	42.5 (39.5, 45.5)	0.72 (0.53, 0.99)	1.13 (1.02, 1.27)	0.54 (0.49, 0.60)	1.35 (1.22, 1.49)
Hospitalizations within the past year								
yes	10.3 (8.7, 11.9)	21.8 (19.6, 24.1)	48.9 (46.3, 51.6)	19.0 (16.7, 21.2)	1.73 (1.45, 2.07)	0.83 (0.74, 0.93)	1.04 (0.98, 1.10)	0.96 (0.84, 1.09)
No	5.6 (5.1, 6.0)	29.8 (29.0, 30.7)	41.9 (40.9, 42.9)	22.7 (21.8, 23.6)	Ref	Ref	Ref	Ref
High-risk condition^a^								
Yes	6.3 (5.6, 7.1)	23.2 (21.9, 24.5)	55.8 (54.3, 57.3)	14.6 (13.5, 15.7)	1.11 (0.95, 1.29)	0.88 (0.82, 0.95)	1.18 (1.13, 1.22)	0.79 (0.73, 0.85)
No	5.8 (5.4, 6.3)	31.2 (30.2, 32.2)	37.8 (36.7, 38.9)	25.1 (24.1, 26.2)	Ref	Ref	Ref	Ref
Previous COVID-19 diagnosis								
Yes	8.8 (7.4, 10.2)	28.3 (26.3, 30.3)	33.7 (31.7, 35.6)	29.2 (27.0, 31.4)	1.39 (1.11, 1.74)	0.93 (0.87, 1.00)	0.85 (0.80, 0.91)	1.23 (1.14, 1.33)
No	5.5 (5.1, 5.9)	29.3 (28.4, 30.1)	44.0 (43.0, 45.0)	21.2 (20.3, 22.1)	Ref	Ref	Ref	Ref
Healthcare provider^b^								
Yes	6.0 (3.8, 8.2)	25.5 (21.5, 29.5)	53.9 (49.4, 58.4)	14.6 (10.9, 18.3)	1.06 (0.71, 1.58)	0.84 (0.72, 0.99)	1.28 (1.17, 1.40)	0.70 (0.54, 0.89)
No	5.8 (5.3, 6.2)	30.0 (29.1, 30.9)	40.8 (39.8, 41.8)	23.4 (22.5, 24.4)	Ref	Ref	Ref	Ref

All percentages are weighted. NH: Non-Hispanic, aPR: adjusted prevalence ratio; CI: confidence intervals; ref: reference

*Adjusted for all variables in the table.

^a^Adults categorized as being at high risk for influenza-related complications reported one or more of the following: (1) ever being told by a physician that they had diabetes, emphysema, chronic obstructive pulmonary disease, coronary heart disease, angina, heart attack, or another heart condition; (2) receiving a diagnosis of cancer during the preceding 12 months (excluding nonmelanoma skin cancer) or ever being told by a physician that they had lymphoma, leukemia, or blood cancer; (3) being told by a physician that they had chronic bronchitis or weak or failing kidneys during the preceding 12 months; or (4) reporting an asthma episode or attack during the preceding 12 months.

^b^Adults were classified as healthcare personnel if they reported that they currently volunteer or work in a hospital, medical clinic, doctor’s office, dentist’s office, nursing home, or some other healthcare facility, including part-time and unpaid work in a healthcare facility as well as professional nursing care provided in the home.

Those who were exclusively vaccinated against COVID-19 were more likely to be 50–64 years (aPR = 1.56) compared to ≥65 years, college graduates (aPR = 1.13) compared to above college graduates, widowed/divorced/separated (aPR = 1.09) or never married (aPR = 1.08) compared to married, and not having a usual place for healthcare (aPR = 1.13) compared to having a usual place for healthcare. Compared to NH White adults, all other races/ethnicities were more likely to be exclusively vaccinated against COVID-19 (NH Black: aPR = 1.24, NH multi-race or other: aPR = 1.22, Hispanic: aPR = 1.28). Females (aPR = 0.90) compared to males, those living in the South (aPR= 0.90) compared to those living in the Northeast, those who had hospitalizations in the past year (aPR = 0.83) compared to those who did not, those who had high-risk conditions (aPR = 0.88) compared to those who did not, and those who were HCPs (aPR = 0.84) compared to those who were not HCPs were less likely to be exclusively vaccinated against COVID-19 (Table 2).

Those who received dual influenza and COVID-19 vaccinations were more likely to be ≥65 years (aPR = 2.24) compared to 18–29 years, female (aPR = 1.09) compared to male, NH multiple race/other (aPR = 1.11) compared to NH White, HCP (aPR = 1.28) compared to non-HCP, had higher than a college degree (aPR = 1.61) compared to high school or less, lived at or above the poverty level (aPR = 1.29) compared to below poverty level, or had a high-risk condition (aPR = 1.18) compared to those who did not. Those who are widowed/divorced/separated (aPR = 0.90) compared to those who were married, did not have health insurance (aPR = 0.43) compared to those who did, did not have a usual place for healthcare (aPR = 0.54) compared to those who did, or had a previous COVID-19 diagnosis (aPR = 0.85) compared to those who did not, were less likely to receive dual vaccinations (Table 2).

Adults who received neither influenza nor COVID-19 vaccinations were more likely to be 18-29 years (aPR = 3.84) compared to ≥65 years, had a high school diploma or less (aPR = 3.65) compared to a higher than a college degree, lived below poverty (aPR = 1.23) compared to at or above poverty, or were widowed/divorced/separated (aPR = 1.20) compared to married. Furthermore, they were more likely to live in the Midwest (aPR = 1.21) or South (aPR = 1.41) compared to the Northeast, did not have health insurance (aPR = 1.29) compared to those who did, did not have a usual place for healthcare (aPR = 1.35) compared to those who did, and had a previous COVID-19 diagnosis (aPR = 1.23) compared to those who did not (Table 2).

Among adolescents, 12–17 years, a large percentage (52.0%) were NH White, lived at or above the poverty level (84.2%), lived in the South (40.6%), had health insurance (95.3%), or had a usual place for healthcare (96.8%) (Table 3). Approximately four percent (3.6%) of adolescents 12-17 years had a high-risk condition or hospitalizations within the past year, and 11.6% had a previous COVID-19 diagnosis.

**Table 3.** Distribution of demographic and other characteristics among parents of children 12–17 years, National Health Interview Survey, United States, 2021.

	Weighted percent (95% CI)
Overall (*n*)	3207
Child’s sex	
Male	51.5 (49.4, 53.5)
Female	48.5 (46.5, 50.6)
Child’s Race/ethnicity	
NH White	52.0 (49.5, 54.5)
NH Black	13.0 (11.2, 14.8)
NH multi/other	9.8 (8.7, 10.9)
Hispanic or Latino	25.2 (22.8, 27.5)
Child’s poverty level	
At or above	84.2 (82.3, 86.1)
Below	15.8 (13.9, 17.7)
Region	
Northeast	15.4 (13.6, 17.1)
Midwest	20.5 (18.6, 22.4)
South	40.6 (38.1, 43.0)
West	23.6 (21.3, 25.9)
Health insurance	
Yes	95.3 (94.4, 96.2)
No	4.7 (3.8, 5.6)
Hospitalizations within the past year	
yes	2.4 (1.7, 3.1)
No	97.6 (96.9, 98.3)
Usual place for healthcare	
Yes	96.8 (96.0, 97.6)
No	3.2 (2.4, 4.0)
High-risk condition*	
Yes	3.6 (2.7, 4.4)
No	96.4 (95.6, 97.3)
Previous COVID-19 diagnosis	
Yes	11.6 (10.2, 12.9)
No	88.4 (87.1, 89.8)

NH: Non-Hispanic; CI: confidence interval.

*High-risk status was defined according to a parental report of 1 or more of the following: ever being told by a physician that the child had diabetes or reporting an asthma episode or attack in the past 12 months. This definition was based on Advisory Committee on Immunization Practices recommendations and the questions available in the NHIS.

Among adolescents, 11.4% were exclusively vaccinated against influenza, 26.4% were vaccinated exclusively against COVID-19, 28.3% were vaccinated against both influenza and COVID-19, and 34.0% were vaccinated against neither (Table 4, Figure 1). Vaccination patterns differed by race/ethnicity, poverty level, region, and hospitalization status. Adolescents who were Hispanic were more likely to be exclusively vaccinated against COVID-19 (aPR = 1.46) compared to adolescents who were NH White. Adolescents who were NH Black were less likely to receive dual vaccinations (aPR = 0.61) than adolescents who were NH White. Adolescents who lived below the poverty level were less likely than adolescents who lived at or above the poverty level to receive exclusive COVID-19 vaccination (aPR = 0.71) or dual vaccination (aPR = 0.60) and more likely to receive neither influenza nor COVID-19 vaccination (aPR = 1.56). Adolescents living in the Midwest (aPR = 1.79) or South (aPR = 1.79) were more likely to receive neither influenza nor COVID-19 vaccination compared to adolescents living in the Northeast. Those who were hospitalized within the past year were more likely (aPR = 1.66) to receive dual vaccination compared to those who were not hospitalized within the past year.

**Table 4.** Bivariate and multivariable analyses of parental report of children’s (12–17 years) receipt of influenza and/or COVID-19 vaccination, National Health Interview Survey, United States, 2021.

	Bivariate analysis				Multivariable analysis			
	Exclusive influenza vaccination	Exclusive COVID-19 vaccination	Dual influenza and COVID-19 vaccination	Neither influenza nor COVID-19 vaccination	Exclusive influenza vaccination	Exclusive COVID-19 vaccination	Dual influenza and COVID-19 vaccination	Neither influenza nor COVID-19 vaccination
	% (95%CI)	% (95%CI)	% (95%CI)	% (95%CI)	aPR (95% CI) *	aPR (95% CI) *	aPR (95% CI) *	aPR (95% CI) *
Overall	11.4 (9.4, 13.4)	26.4 (23.7, 29.0)	28.3 (25.6, 30.9)	34.0 (30.9, 37.1)				
Child’s sex								
Male	12.9 (10.1, 15.8)	25.8 (22.3, 29.3)	28.1 (24.6, 31.6)	33.2 (29.1, 37.3)	Ref	Ref	Ref	Ref
Female	9.7 (7.1, 12.3)	27.0 (23.2, 30.7)	28.5 (24.8, 32.2)	34.8 (30.5, 39.2)	0.75 (0.55, 1.03)	1.04 (0.86, 1.25)	1.00 (0.84, 1.19)	1.07 (0.92, 1.26)
Child’s Race/ethnicity								
NH White	11.0 (8.4, 13.6)	23.1 (19.7, 26.4)	31.7 (28.0, 35.4)	34.2 (30.1, 38.3)	Ref	Ref	Ref	Ref
NH Black	13.5 (7.5, 19.6)	28.4 (20.0, 36.8)	18.2 (11.6, 24.8)	39.9 (31.2, 48.7)	1.12 (0.70, 1.81)	1.35 (0.98, 1.87)	0.61 (0.42, 0.90)	1.04 (0.78, 1.38)
NH multi/other	12.4 (6.7, 18.0)	27.0 (20.5, 33.5)	36.7 (28.8, 44.6)	24.0 (16.0, 31.9)	1.15 (0.71, 1.86)	1.14 (0.84, 1.54)	1.16 (0.91, 1.47)	0.72 (0.49, 1.05)
Hispanic or Latino	10.8 (7.0, 14.6)	32.2 (26.5, 37.9)	22.8 (18.3, 27.3)	34.2 (28.2, 40.1)	0.92 (0.58, 1.44)	1.46 (1.21, 1.76)	0.79 (0.63, 1.00)	0.91 (0.75, 1.11)
Child’s Poverty level								
At or above (ref)	11.1 (8.9, 13.2)	27.2 (24.5, 30.0)	30.2 (27.0, 3, 33)	31.6 (28.4, 34.7)	Ref	Ref	Ref	Ref
Below	13.2 (8.0, 18.5)	21.3 (14.5, 28.2)	17.2 (11.8, 22.6)	48.2 (39.4, 56.9)	1.28 (0.86, 1.91)	0.71 (0.53, 0.96)	0.60 (0.42, 0.85)	1.56 (1.26, 1.94)
Region								
Northeast	12.6 (7.1, 18.0)	32.0 (24.0, 39.9)	34.2 (26.1, 42.4)	21.3 (13.2, 29.3)	Ref	Ref	Ref	Ref
Midwest	10.7 (7.1, 14.4)	22.8 (17.4, 28.1)	29.5 (23.7, 35.2)	37.0 (30.3, 43.8)	0.84 (0.48, 1.48)	0.75 (0.51, 1.09)	0.82 (0.57, 1.17)	1.79 (1.21, 2.65)
South	12.5 (9.5, 15.5)	24.7 (20.5, 28.8)	23.7 (19.9, 27.5)	39.1 (34.0, 44.2)	0.95 (0.55, 1.62)	0.74 (0.55, 0.99)	0.78 (0.58, 1.05)	1.79 (1.21, 2.64)
West	9.3 (4.4, 14.2)	28.9 (23.5, 34.4)	31.3 (25.5, 37.1)	30.5 (24.4, 36.5)	0.76 (0.35, 1.67)	0.87 (0.63, 1.20)	0.94 (0.67, 1.31)	1.44 (0.95, 2.19)
Health insurance								
Yes	11.3 (9.2, 13.3)	26.3 (23.6, 29.0)	29.0 (26.3, 31.8)	33.4 (30.3, 36.5)	Ref	Ref	Ref	Ref
No	15.5 (5.8, 25.1)	31.4 (17.7, 45.1)	9.0 (5.0, 13.1)	44.0 (28.5, 59.5)	1.15 (0.49, 2.69)	1.16 (0.78, 1.72)	0.42 (0.21, 0.82)	1.18 (0.91, 1.52)
Hospitalizations within the past year								
Yes	6.4 (0.9, 11.9)	23.0 (13.4, 32.6)	43.6 (29.5, 57.6)	27.0 (12.0, 42.0)	0.57 (0.20, 1.62)	0.92 (0.49, 1.73)	1.66 (1.17, 2.35)	0.73 (0.42, 1.25)
No	11.6 (9.5, 13.6)	26.5 (23.8, 29.1)	27.8 (25.0, 30.5)	34.2 (31.0, 37.4)	Ref	Ref	Ref	Ref
Usual place for healthcare								
Yes	11.1 (9.1, 13.1)	26.4 (23.7, 29.1)	28.9 (26.1, 31.6)	33.6 (30.5, 36.7)	Ref	Ref	Ref	Ref
No	20.5 (0.1, 40.9)	24.3 (14.3, 34.4)	9.6 (1.5, 17.8)	45.5 (26.0, 65.1)	1.80 (0.69, 4.70)	0.83 (0.47, 1.47)	0.44 (0.21, 0.91)	1.25 (0.84, 1.86)
High-risk condition^a^								
Yes	12.3 (3.2, 21.4)	13.4 (1.4, 25.4)	34.2 (22.8, 45.6)	40.1 (26.2, 54.1)	1.08 (0.51, 2.29)	0.53 (0.23, 1.20)	1.32 (0.87, 1.98)	1.07 (0.73, 1.57)
No	11.3 (9.3, 13.4)	26.9 (24.2, 29.6)	28.0 (25.3, 30.8)	33.7 (30.6, 36.9)	Ref	Ref	Ref	Ref
Previous COVID-19 diagnosis								
Yes	15.5 (9.3, 21.6)	24.4 (18.0, 30.7)	21.5 (15.3, 27.7)	38.7 (29.8, 47.5)	1.45 (0.91, 2.31)	0.91 (0.69, 1.22)	0.69 (0.53, 0.90)	1.23 (0.95, 1.58)
No	10.8 (8.7, 12.9)	26.7 (23.9, 29.4)	29.3 (26.4, 32.2)	33.3 (30.1, 36.4)	Ref	Ref	Ref	Ref

All percentages are weighted. NH: Non-Hispanic; aPR: adjusted prevalence ratio; CI: confidence intervals; ref: reference. *Adjusted for all variables in the table.

^a^High-risk status was defined according to a parental report of 1 or more of the following: ever being told by a physician that the child had diabetes or reporting an asthma episode or attack in the past 12 months. This definition was based on Advisory Committee on Immunization Practices recommendations and the questions available in the NHIS.

## Discussion

With the possibility of a future “twindemic” or “tripledemic” caused by high cases of COVID-19, influenza, or other respiratory viruses that could overwhelm healthcare systems and resources, it is critical for all eligible people to receive the primary COVID-19 vaccines series, updated COVID-19 booster, and annual influenza vaccines. This study found that only 42.5% of adults and 28.3% of adolescents ages 12–17 years have received both influenza and COVID-19 vaccines, while approximately a quarter (22.4%) of adults and a third (34.0%) of adolescents did not receive either vaccine. While COVID-19 vaccination coverage (≥1 dose) is much higher now at 92.1% among adults ≥18 years and 72.0% among adolescents 12–17 years as of March 2023, the updated (bivalent) booster vaccination coverage is only 19.7% among adults and 7.3% among adolescents 12–17 years during the same time period [26]. Using the National Immunization Survey, the CDC also found that influenza coverage among adults was 21.2% as of 9 October 2022, and 24.8% for adolescents 6 months to 17 years as of 22 October 2022 [27]. For adolescents, these estimates are the same compared to the same time in October 2021 but 7.3 percentage points lower compared with October 2020 [27]. These estimates suggest that more efforts are needed to increase influenza and COVID-19 booster vaccination among adolescents and adults in 2023 and beyond.

Patterns of vaccination differed by sociodemographic and other characteristics. For example, adults who received exclusive COVID-19 or dual vaccination were more likely to be similar in terms of age, race/ethnicity, and educational attainment. With the exception of those aged ≥65 years and older, exclusive COVID-19 or dual vaccination increased with increasing age, was highest among NH multi/other races, and those with higher educational attainment compared to their respective counterparts. Adults who received exclusive influenza vaccination or neither influenza nor COVID-19 vaccination were also similar. Adults in this group were more likely to be younger, had a high school diploma or less, lived below the poverty level, and had a previous COVID-19 diagnosis compared to their respective counterparts. Adults and adolescents who lived in the South and Midwest were more likely to receive neither vaccine than those living in the Northeast. Those who received exclusive COVID-19 or dual vaccinations represented a larger proportion of adults (71.6%) while those who received exclusive influenza or neither vaccination represented 28.4% of adults. These results can be used to tailor messages and interventions for groups with the largest disparities in vaccination coverage. For example, the CDC and other health experts recommend that effective messages, or those that have undergone testing with the intended population, are provided by trusted messengers and vaccine ambassadors [22,23]. These are people who are seen as credible sources of information by specific populations, such as doctors, community leaders, or religious leaders. Messages should increase people’s confidence in vaccines and address mistrust and misinformation [23,28].

While HCP were more likely to receive dual vaccinations than adults who were not HCP, dual vaccination coverage among HCP was 53.9% in 2021, despite this group being prioritized and recommended for the COVID-19 vaccine [2]. While coverage may be higher now due to increased availability of COVID-19 vaccines and possible workplace mandates [29–31], it is important for HCP to receive the completed primary series and updated booster for COVID-19, in addition to the annual influenza vaccine, and if appropriate, to recommend vaccines to their patients. Studies have found that healthcare provider recommendations for COVID-19 vaccines increased vaccination uptake as well as vaccine confidence in adults [22,32].

Access to care factors such as having health insurance and a usual place for healthcare were significant predictors of dual vaccination for both adults and adolescents. As a result, ensuring that adolescents and adults have access to healthcare, such as a primary care doctor, is essential for increasing vaccination uptake [22]. Although the ACIP recommends that adults and adolescents with high-risk conditions receive influenza and COVID-19 vaccinations, our study found that adults, but not adolescents, with high-risk conditions were more likely to receive dual vaccinations than those without high-risk conditions. Interestingly, adults with a previous COVID-19 diagnosis were less likely to receive exclusive COVID-19 or dual vaccination and were more likely to receive neither influenza nor COVID-19 vaccine than adults without a previous COVID-19 diagnosis. In addition, adolescents with a prior COVID-19 diagnosis were less likely to receive dual vaccination than adolescents without a prior COVID-19 diagnosis. This is consistent with other studies showing that those with prior COVID-19 diagnosis were less likely to receive a COVID-19 vaccination, due to the false belief that they were already protected against future infections [33]. Eligible adults and adolescents, even those with high-risk or prior COVID-19 diagnosis, should receive all updated doses of COVID-19 vaccinations to protect themselves from severe health outcomes. Ensuring access to healthcare resources and promoting confidence among HCP to get vaccinated and recommend vaccination to their patients, is needed to bolster vaccination uptake among adults and adolescents [21].

The study is subject to a few limitations. First, influenza and COVID-19 vaccination status were based on self-reported data (for adults) and parental reports (for adolescents) and were not verified by medical records, so may be subject to recall or social-desirability bias. Second, high-risk medical conditions reported in this study were not inclusive of all high-risk conditions for influenza and COVID-19; rather they were limited by questions available in the survey and were also self-reported so may be subject to misclassification. Third, COVID-19 vaccination coverage may be under-reported if adolescents or adults were not eligible for the vaccine at the time of the survey, and the association between factors associated with exclusive or dual COVID-19 vaccination may be greater than presented in this study. Fourth, COVID-19 questions were only added starting in 2021, so assessment of vaccination patterns by seasons prior to 2021 is not possible. Finally, survey completion response rates were 50.9% for adults and 49.9% for adolescents, and vaccination status may differ between survey respondents and nonrespondents; survey weighting adjustments may not adequately control for these differences.

## Conclusion

During the COVID-19 pandemic, approximately two-thirds and three-fourths of adolescents and adults, respectively, received either influenza and/or COVID-19 vaccines in 2021. Vaccination patterns differed by sociodemographic and access to care characteristics, with those who had lower educational attainment or income, or no health insurance, more likely to have received neither vaccination compared to those with higher educational attainment or income or those with health insurance, respectively. Furthermore, those who lived in the South or Midwest were more likely to receive neither vaccination compared to those who lived in the Northeast. These disparities highlight possible to access or logistical barriers to these vaccines, or vaccine hesitancy. Coadministration of influenza and COVID-19 vaccinations could increase the probability of people being up to date on recommended vaccines [34]. Understanding gaps in coverage is needed to develop appropriate messages and strategies to increase vaccine uptake. Presently, COVID-19 and influenza vaccines are recommended for all adults and children ≥6 months, and updated COVID-19 boosters are recommended for children ≥5 years. Promoting confidence in vaccines and reducing barriers to access is needed to protect individuals and families from severe health consequences of vaccine-preventable diseases. Being up-to-date with all recommended vaccinations can prevent a future resurgence of hospitalizations and cases, especially as new variants emerge.

## Data Availability

The data that support the findings of this study are openly available at https://www.cdc.gov/nchs/nhis/data-questionnaires-documentation.htm

## References

[1] CDC [Internet]. Influenza ACIP vaccine recommendations. Available from: https://www.cdc.gov/vaccines/hcp/acip-recs/vacc-specific/flu.html.

[2] CDC [Internet]. COVID-19 ACIP vaccine recommendations. Available from: https://www.cdc.gov/vaccines/hcp/acip-recs/vacc-specific/covid-19.html.

[3] Rubin R. The dreaded "twindemic" of influenza and COVID-19 has not yet materialized-might this be the year? JAMA.2022;328(15):1–11. 10.1001/jama.2022.15062 36129724

[4] Grohskopf LA, Liburd LC, Redfield RR. Addressing influenza vaccination disparities during the COVID-19 pandemic. JAMA. 2020;324(11):1029–1030. 32930764 10.1001/jama.2020.15845 PMC10926999

[5] Schmid P, Rauber D, Betsch C, et al. Barriers of influenza vaccination intention and behavior - A systematic review of influenza vaccine hesitancy, 2005 – 2016. PLoS One. 2017;12(1):e0170550. 28125629 10.1371/journal.pone.0170550 PMC5268454

[6] Lu PJ, Hung MC, Srivastav A, et al. Surveillance of vaccination coverage among adult populations – United States, 2018. MMWR Surveill Summ. 2021;70(3):1–26. 10.15585/mmwr.ss7003a1 PMC8162796 33983910

[7] Santibanez TA, Srivastav A, Zhai Y, et al. Trends in childhood influenza vaccination coverage, United States, 2012–2019. Public Health Rep. 2020;135(5):640–649. 32783780 10.1177/0033354920944867 PMC7485062

[8] Santibanez TA, Kennedy ED. Reasons given for not receiving an influenza vaccination, 2011–12 influenza season, United States. Vaccine. 2016;34(24):2671–2678. 27118168 10.1016/j.vaccine.2016.04.039 PMC5751433

[9] Lu PJ, O’Halloran A, Bryan L, et al. Trends in racial/ethnic disparities in influenza vaccination coverage among adults during the 2007–08 through 2011–12 seasons. Am J Infect Control. 2014;42(7):763–769. 24799120 10.1016/j.ajic.2014.03.021 PMC5822446

[10] Lu PJ, Hung MC, O’Halloran AC, et al. Seasonal influenza vaccination coverage trends among adult populations, U.S., 2010-2016. Am J Prev Med. 2019;57(4):458–469. 31473066 10.1016/j.amepre.2019.04.007 PMC6755034

[11] Callaghan T, Moghtaderi A, Lueck JA, et al. Correlates and disparities of intention to vaccinate against COVID-19. Soc Sci Med. 2021;272:113638. 33414032 10.1016/j.socscimed.2020.113638 PMC7834845

[12] Williams AM, Clayton HB, Singleton JA. Racial and ethnic disparities in COVID-19 vaccination coverage: the contribution of socioeconomic and demographic factors. Am J Prev Med. 2022;62(4):473–482. 34872772 10.1016/j.amepre.2021.10.008 PMC8598940

[13] Valier MR, Elam-Evans LD, Mu Y, et al. Racial and ethnic differences in COVID-19 vaccination coverage among children and adolescents aged 5–17 years and parental intent to vaccinate their Children – National immunization survey-child COVID module, United States, december 2020-September 2022. MMWR Morb Mortal Wkly Rep. 2023;72(1):1–8. 36602930 10.15585/mmwr.mm7201a1 PMC9815155

[14] Goralnick E, Kaufmann C, Gawande AA. Mass-vaccination sites – an essential innovation to curb the Covid-19 pandemic. N Engl J Med. 2021;384(18):e67. 33691058 10.1056/NEJMp2102535

[15] CDC [Internet]. COVID-19 Vaccinations in the United States. Avialble from: https://covid.cdc.gov/covid-data-tracker/#vaccinations_vacc-total-admin-rate-total.

[16] Nguyen KH, Nguyen K, Mansfield K, et al. Child and adolescent COVID-19 vaccination status and reasons for non-vaccination by parental vaccination status. Public Health. 2022;209:82–89. 35870290 10.1016/j.puhe.2022.06.002 PMC9189141

[17] Olusanya OA, Bednarczyk RA, Davis RL, et al. Addressing parental vaccine hesitancy and other barriers to childhood/adolescent vaccination uptake during the coronavirus (COVID-19) pandemic. Front Immunol. 2021;12:663074. 33815424 10.3389/fimmu.2021.663074 PMC8012526

[18] Middleman AB, Klein J, Quinn J. Vaccine hesitancy in the time of COVID-19: attitudes and intentions of teens and parents regarding the COVID-19 vaccine. Vaccines. 2021;10(1):4. 10.3390/vaccines10010004 PMC8777704 35062665

[19] Tewarson H, Greene K, Fraser MR. State strategies for addressing barriers during the early US COVID-19 vaccination campaign. Am J Public Health. 2021;111(6):1073–1077. 33950715 10.2105/AJPH.2021.306241 PMC8101562

[20] Zhang Y, Fisk RJ. Barriers to vaccination for coronavirus disease 2019 (COVID-19) control: experience from the United States. Glob Health J. 2021;5(1):51–55. 33585053 10.1016/j.glohj.2021.02.005 PMC7871809

[21] CDC [Internet]. Vaccinate with confidence COVID-19 vaccines strategy for adults. Available from: https://www.cdc.gov/vaccines/covid-19/vaccinate-with-confidence/strategy.html.

[22] CDC [Internet]. 12 COVID-19 vaccination strategies for your community. Available from: https://www.cdc.gov/vaccines/covid-19/vaccinate-with-confidence/community.html.

[23] Academies N. Communication strategies for promoting COVID-19 vaccine acceptance Available from: https://nap.nationalacademies.org/resource/26068/interactive/vaccine-confidence.html.

[24] Statistics NCfH [Internet]. 2021. Survey description. https://ftp.cdc.gov/pub/Health_Statistics/NCHS/Dataset_Documentation/NHIS/2021/srvydesc-508.pdf.

[25] Fiore AE, Uyeki TM, Broder K, et al. Prevention and control of influenza with vaccines: recommendations of the advisory committee on immunization practices (ACIP), 2010. MMWR Recomm Rep. 2010;59(RR-8):1–62. 20689501

[26] CDC [Internet]. Trends in number of COVID-19 vaccinations in the US. Centers for Disease Control and Prevention. [cited on 2022 June 10]. https://covid.cdc.gov/covid-data-tracker/#vaccination-trends.

[27] CDC [Internet]. Weekly flu vaccination dashboard. Available from: https://www.cdc.gov/flu/fluvaxview/dashboard/vaccination-dashboard.html.

[28] CDC [Internet]. Vaccinate with confidence. Available from: https://www.cdc.gov/vaccines/partners/vaccinate-with-confidence.html.

[29] Vaccine MANDATES by state: Who is, who isn’t, and how [Internet]? Leading age. Available from: https://leadingage.org/vaccine-mandates-by-state-who-is-who-isnt-and-how/.

[30] Razzaghi H, Masalovich S, Srivastav A, et al. COVID-19 vaccination and intent among healthcare personnel, U.S. Am J Prev Med. May 2022;62(5):705–715. 34965901 10.1016/j.amepre.2021.11.001 PMC8710229

[31] Lee JT, Sean Hu S, Zhou T, et al. Employer requirements and COVID-19 vaccination and attitudes among healthcare personnel in the U.S.: findings from national immunization survey adult COVID module, August – September 2021. Vaccine. 2022;40(51):7476–7482. 35941037 10.1016/j.vaccine.2022.06.069 PMC9234000

[32] Nguyen KH, Yankey D, Lu PJ, et al. Report of health care provider recommendation for COVID-19 vaccination among adults, by recipient COVID-19 vaccination status and attitudes – United States, April–September 2021. MMWR Morb Mortal Wkly Rep. 2021;70(50):1723–1730. 34914669 10.15585/mmwr.mm7050a1 PMC8675662

[33] Nguyen KH, Huang J, Mansfield K, et al. COVID-19 vaccination coverage, behaviors, and intentions among adults with previous diagnosis, United States. Emerg Infect Dis. 2022;28(3):631–638. 35202522 10.3201/eid2803.211561 PMC8888235

[34] Association AM [Internet]. What to know about coadministration of flu and COVID-19 vaccines. Available from: https://www.ama-assn.org/delivering-care/public-health/what-know-about-coadministration-flu-and-covid-19-vaccines.

